# Targeting the Wnt pathways for therapies

**DOI:** 10.1186/2052-8426-2-28

**Published:** 2014-09-11

**Authors:** Artem Blagodatski, Dmitry Poteryaev, Vladimir L Katanaev

**Affiliations:** Institute of Protein Research, Russian Academy of Sciences, Pushchino, Russian Federation; IBC Generium LLC, Volginsky, Russian Federation; Department of Pharmacology and Toxicology, University of Lausanne, Lausanne, Switzerland

**Keywords:** Wnt, Frizzled, Cancer, Regeneration, Drug discovery

## Abstract

The Wnt/β-catenin signaling pathway is crucial in animal development from sponges to humans. Its activity in the adulthood is less general, with exceptions having huge medical importance. Namely, improper activation of this pathway is carcinogenic in many tissues, most notably in the colon, liver and the breast. On the other hand, the Wnt/β-catenin signaling must be re-activated in cases of tissue damage, and insufficient activation results in regeneration failure and degeneration. These both medically important implications are unified by the emerging importance of this signaling pathway in the control of proliferation of various types of stem cells, crucial for tissue regeneration and, in case of cancer stem cells – cancer progression and relapse. This article aims at briefly reviewing the current state of knowledge in the field of Wnt signaling, followed by a detailed discussion of current medical developments targeting distinct branches of the Wnt pathway for anti-cancer and pro-regeneration therapies.

## Introduction: the Wnt signaling pathways

**Figure 1:**
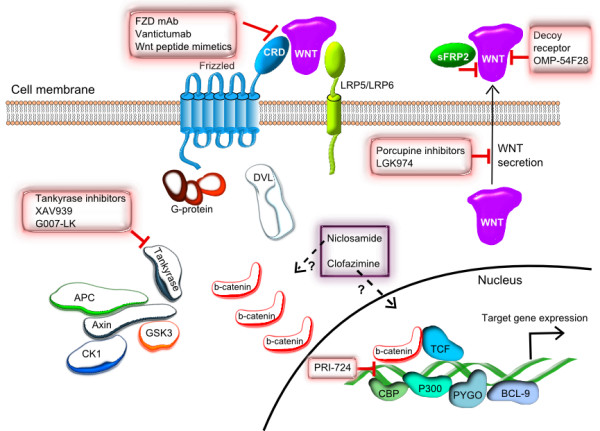
**Schematic representation of the Wnt/β-catenin signaling pathway and the oncology-indication drug candidates discussed in the paper.** The molecular targets (where known) of the small molecule and antibody-based drug candidates are shown.

The Wnt signaling plays instrumental roles in animal development [1]. This type of intracellular signaling apparently was ‘invented’ together with (or in requirement for) animal multi-cellularity, as its architecture and components are already present in the simplest metazoans such as sponges and ctenophores [2, 3]. The pathway is initiated by secreted lipoglycoproteins of the Wnt family, of which 19 members exist in humans. Given the numerous post-translational modifications and the need for a tightly controlled manner of Wnt diffusion through the tissue, the secretion apparatus within the Wnt-producing cells is rather complex [4]. In the signal-receiving cells, Wnt activates a receptor of the Frizzled (FZD) family (10 members in humans) and a LRP5/6 co-receptor (2 members in humans). While LRP5/6 are single-transmembrane proteins, FZDs possess seven transmembrane domains and belong to the G protein-coupled receptor (GPCR) superfamily [5]. Although initially questioned as functional GPCRs, FZDs in recent years have been clearly demonstrated, by genetic and biochemical means, to signal through heterotrimeric G proteins [6–11]. Together with the latter, a cytoplasmic protein Dishevelled (Dvl) acts as an immediate transducer of the signal from the receptors [12]. Both types of transducers act on Axin [13, 14] – a key component of the β-catenin-destruction complex also including the protein APC and kinases GSK3β and casein kinase. The function of this complex is to phosphorylate cytoplasmic β-catenin, targeting it for ubiquitin-dependent proteasomal degradation [15]. With dysfunctional Axin, the destruction complex becomes inactive, leading to accumulation of β-catenin, its translocation to the nucleus, and activation of LEF/TCF-dependent transcription [16] (Figure 1). Among Wnt-target genes are pro-proliferative c-Myc and cycD1 [17]. In addition to the pathway directly initiated from the plasma membrane, Wnt-receptor complexes can also be internalized, in a heterotrimeric G protein- and Rab5-dependent manner, into signaling endosomes, which is required for the amplification of the signaling strength [18, 19].

The β-catenin-dependent Wnt signaling pathway described above is often referred to as the "canonical" Wnt signaling. In addition to it, a group of "non-canonical", β-catenin-independent pathways can be initiated by Wnt/FZD complexes [20]. They can increase concentration of intracellular calcium ions and regulate the cytoskeleton and ultimately – cell polarity and motility. They can also antagonize the β-catenin-dependent signaling in certain contexts [21]. Apart from heterotrimeric G proteins and Dvl, no other components of the β-catenin-dependent pathway are involved in "non-canonical" signaling. Our review will only episodically touch upon these types of Wnt signaling, mostly concentrating on the Wnt/β-catenin pro-proliferative branch.

The Wnt/β-catenin signaling is repeatedly used during animal development. Given this important developmental function, the role of this pathway in regulation of stem cell proliferation and differentiation, which emerges as a unifying function of this pathway in the adult, both in the physiological and pathological contexts, is not surprising. Initially Wnt/β-catenin signaling has been shown to be crucial for the maintenance and self-renewal of hematopoietic cells [22]. This discovery was corroborated by subsequent findings of the necessity of this pathway for proliferation of neuronal [23], embryonic [24], mammary [25], intestinal [26] and other types of stem cells. Finally, the role of Wnt/β-catenin signaling in cancer stem cells (CSCs) has also emerged [27]. These discoveries form the basis for the translational efforts of targeting (suppressing or activating) the Wnt/β-catenin signaling in anti-cancer and regeneration therapies, discussed below.

## Review

### Development of anti-cancer drugs targeting the Wnt/β-catenin pathway

The Wnt/β-catenin signaling pathway as a target of anti-cancer drugs has attracted attention of biotech companies relatively recently. This field received a special attention when it became clear that this signaling plays a major role in CSC proliferation. CSCs have been implicated in tumor maintenance and relapse after surgical resection. The CSC pool is self-renewing and this process is largely driven by re-activation of embryonic programs mediated by Wnt, Hedgehog and Notch signaling pathways and the mTOR signaling hub [27, 28].

A large number of preclinical experiments demonstrated that inhibition of Wnt/β-catenin signaling can affect cancer cell growth and survival (for review see [29]). While mutations in the Wnt/β-catenin pathway are responsible for certain types of cancers, most notably APC mutations in colorectal cancer [30], many cancers driven by overstimulation of this signaling do not harbor mutations in its components. For example, the study of 245 invasive breast carcinomas has identified a subgroup with triple-negative phenotype (ER-, PgR-, HER2-) where β-catenin was accumulated in the nucleus, which is a hallmark of Wnt/β-catenin pathway activity. However, no β-catenin mutation has been found in all triple-negative carcinomas analyzed [31]. Therefore constitutive receptor stimulation can account for hyperactive Wnt signaling in the absence of activating mutations in the components of the pathway.

Most current anti-cancer drugs, small molecule inhibitors and monoclonal antibodies (mAbs), are designed to target rapidly proliferating cells which represent committed cancer cells but not CSCs. Sorafenib, a small molecule inhibitor of multiple tyrosine kinases involved in tumor proliferation, is used in the treatment of acute myeloid leukemia (AML). There it is supposed to inhibit FMS-like kinase overexpressed in almost all cases of AML. As has been recently demonstrated, sorafenib effectively reduces the number of mature AML progenitors but fails to eradicate AML stem cells and primitive progenitors [32]. Trastuzumab is an example of a mAb. It targets the HER2 receptor overexpressed in one quarter of breast cancers. Trastuzumab monotherapy has only a 30% response rate and acquired resistance to trastuzumab occurs frequently. Trastuzumab has been shown to be effective only in the context of PI3K signaling and in the presence of PTEN, but CSCs display the aberrant former and the absence of latter [33]. Since current cancer therapies fail to eradicate CSCs, selective targeting of CSCs would be a promising therapeutic strategy.

### Small molecules targeting the Wnt/β-catenin pathway

Significant efforts are made world-wide to develop potent inhibitors of the Wnt/β-catenin signaling, but only few of these have made it to reach clinical trials. Small-molecule inhibitors identified in a number of high-throughput screens can be classified into four groups: β-catenin/TCF-antagonists, modulators of transcription co-activator, Dvl binders, and other mechanism-based inhibitors [34] (Figure 1 and Table 1).

**Table 1:** **Current status of clinical trials of biologics specifically targeting the Wnt/β-catenin pathway (ligands or receptors)**

Name/company	Target	Agent	Conditions	Clinical phase
OTSA101 (Centre Léon Bérard, OncoTherapy Science)	FZD_10_	mAb	Synovial sarcoma. Antibody-radionuclide conjugate (^90^Y)	I
OMP-54F28 (Oncomed Pharma)	Wnt	Fzd8-Fc (scavenging receptor)	HCC, liver cancer, ovarian cancer, pancreatic cancer, other solid tumors	I
Vantictumab (Oncomed Pharma)	FZD_1_, _2_, _5_, _7_, _8_	mAb	Solid tumors (completed), NSCLC, metastatic breast cancer, pancreatic cancer, (active, as a combination with chemiotherapy)	I
Foxy-5 (WntResearch AB)	FZD_5_	Peptide	Metastatic breast cancer, colorectal cancer, prostate cancer	I

Among small molecules the leading class is the Porcupine inhibitors, as exemplified by LGK974 (Novartis) [35]. Porcupine is a membrane-bound O-acetyltransferase required for acylation of Wnt molecules, and inhibition of this enzyme results in reduced or abolished Wnt secretion [36]. LGK974 is now in clinical Phase I for the following conditions: melanoma, breast cancer (triple-negative), pancreatic adenocarcinoma, colorectal cancer, head and neck cancers. Additionally patients with other tumor types with documented genetic alterations upstream in the Wnt/β-catenin signaling pathway are being recruited ( http://www.clinicaltrials.gov).

Another class of compounds inhibits tankyrases (TNK1 and 2) – enzymes among other functions found to destabilize Axin. TNK inhibition results in prevention of β-catenin accumulation. Examples are XAV939 from Novartis [37] and G007-LK from Roche [38]. The active development of TNK inhibitors is pursued for two reasons: first, Axin is the rate-limiting component of the β-catenin destruction complex [39]; second, Axin mutations and increased β-catenin levels are associated with various types of cancer [40]. In cancers with mutated Axin or APC, the upstream antagonists acting on Wnts or their receptors may be less effective. However, the full potential of the antitumor activity of TNK inhibitors may be limited by intestinal toxicity associated with inhibition of Wnt/β-catenin signaling and cell proliferation in intestinal crypts [38].

During activation of β-catenin-dependent gene transcription, the complex of β-catenin and LEF/TCF recruits additional factors for chromatin remodeling, like CBP and p300, which possess the histone acetyltransferase activity. Association of β-catenin with histone acetylases can be antagonized by several compounds. One of them, PRI-724 (Prism Pharma) has reached clinical trials in AML and advanced solid tumors. It is a small molecule that selectively inhibits the histone deacetylase CBP/β-catenin complex, blocking expression of the Wnt/β-catenin pathway-dependent pro-growth and pro-survival genes. PRI-724 exhibits a selective antiproliferative effect, inhibiting various cancer cell lines *in vitro* and substantially inhibiting tumor growth in animal studies ( http://clinicaltrials.gov/show/NCT01302405).

Already approved drugs with well-established safety profile find a far easier way to clinical trials for a different indication. Niclosamide is an anti-helminthic drug used in humans for nearly 50 years. In 2009, it emerged as a compound that inhibits Wnt3a-stimulated β-catenin stabilization and TCF/LEF reporter activity in osteosarcoma cell line. This was a result of screening of an FDA-approved drug library for compounds that would promote endocytosis of FZD_1_ [41]. *In vitro*, niclosamide treatment reduced the levels of LRP6 and β-catenin, and *in vivo* it had a suppressive effect on basal breast cancer xenografts [42, 43]. Despite these observations, niclosamide is not ready yet for clinical trials for oncology indications. As an approved drug it is given orally and is only partially absorbed from the gastrointestinal tract, therefore novel derivatives are needed to improve the bioavailability of niclosamide. The alternative intravenous route of niclosamide administration requires comprehensive investigation regarding the safety and the possibility of systemic application [43]. Other potential anti-Wnt drug candidates emerge from screening of FDA-approved compounds; the anti-leprosy drug clofazimine has recently been discovered as a potent inhibitor of Wnt/β-catenin signaling and proliferation of Wnt-dependent triple negative breast cancer cells [44].

### Wnts as targets

Although there has been a number of reports where Wnt proteins were targeted directly by antibodies (see for example [45, 46]), none of the anti-Wnt mAbs is currently visible even in the pre-clinical pipelines of pharma companies. Another way to neutralize Wnt ligands is chosen by the company OncoMed Pharmaceuticals. Its candidate biologic OMP-54F28 is a fusion between the Wnt-binding CRD domain of FZD8 and the Fc-fragment of IgG. OMP-54F28 works as a scavenger for Wnt proteins (apparently several of the family) preventing them from binding to endogenous membrane-bound FZDs [47]. Surprisingly, despite pronounced reduction of xenograft tumor growth in mice, OMP-54F28 treatment did not visibly change the levels or cellular localization of β-catenin in xenograft tissues. This suggests that although changes in β-catenin may have been too small to detect by immunohistochemistry, the attenuation of Wnt/β-catenin signaling was sufficient to inhibit the tumor growth. More importantly, this study has shown no adverse effects in the skin and intestine ( http://www.oncomed.com/Pipeline.html) (but see section "Safety of Wnt pathway targeting" below).

### FZDs as targets

The FZD family of GPCRs provides a large and practically untapped source of potential targets for therapeutic interventions [48]. A number of pharma companies are searching for novel GPCR-interacting molecules. The most high-throughput approach is the screening of small molecule chemical libraries to identify candidate therapeutics. Yet, in the past decade the number of small molecules targeting GPCRs that were approved as therapeutics has been very low. The high attrition rate in preclinical and clinical studies, credited to toxicity, low efficacy or selectivity puts an enormous burden on drug discovery budgets. In contrast to that, protein biologics, such as monoclonal antibodies (mAbs), have several advantages as therapeutics. They are highly selective and have much longer half-lives than small molecules [49, 50].

Peptide fragments of Wnt ligands, binding to the CRD domain of FZD receptors, have been proposed as potential therapeutic agents. Indeed, *in vitro* experiments indicate that these peptides can compete with full-length Wnts and attenuate canonical signaling. However one can doubt their value even in animal model preclinical studies, since the rapid clearance of non-modified peptides would prevent any lasting effect on cancer cells. Such antagonist mimetics of Wnts would need to be modified, for example by PEGylation or formylation, to effectively increase their half-life, before considering them as therapeutic candidates. A hexapeptide Box5, derived from Wnt5a and stabilized by the N-butyloxycarbonyl group, has been developed to antagonize Wnt5a-stimulated metastasis in melanoma [51]. In contrast to its activity in melanoma, Wnt5a shows tumor-suppressing activity in the breast, and restoring this protein can suppress migration of breast cancer cells – activity recapitulated by a formylated hexapeptide Foxy-5 also derived from Wnt5a [52, 53]; this drug candidate is currently in phase I clinical trials.

FZD_10_ has a very restricted expression pattern; it is undetectable in normal human tissues except placenta, but up-regulated in synovial sarcomas. Taking this opportunity, OncoTherapy Science has developed a chimeric humanized mAb against FZD_10_, named OTSA101. Non-radiolabeled OTSA101 antibody has only weak antagonistic activity on synovial sarcoma cell growth. However, Yttrium 90-radiolabeled OTSA101 (OTSA101-DTPA-^90^Y) showed significant antitumor activity following a single intravenous injection in mouse xenograft model [54] and is now in Phase I clinical studies.

FZD_7_ is the Wnt receptor most commonly up-regulated in a variety of cancers including colorectal cancer, hepatocellular carcinoma (HCC) and triple negative breast cancer [55]. It has been demonstrated that siRNA (small interfering RNA) knockdown of FZD_7_ displayed anti-cancer activity *in vitro* and *in vivo* due to the inhibition of the canonical Wnt signaling pathway [56]. While siRNA approach remains to be problematic in clinic for a number of reasons, anti-FZD mAb named vantictumab is one of the lead protein biologics in the Oncomed Pharmaceuticals pipeline for oncology indications. This antibody was initially identified by binding to FZD_7_’ CRD domain and ability to inhibit Wnt3a-induced signaling in a cell-based assay. In addition to FZD_7_, vantictumab also binds to four other FZD receptors (out of ten encoded by the human genome) – restricting its selectivity (see below). In mouse xenograft models of several types of solid tumors (among them pancreatic, colon cancers, and triple-negative breast cancer), vantictumab as a monotherapy demonstrated just a decrease in the tumor growth rate. On the other hand, combined treatment with cytotoxic chemotherapy produced in some tumor types not only a dramatic tumor shrinkage but also tumor cell differentiation and reduction of CSC numbers [57]. The interim data of vantictumab clinical trials (Phase I) have been reported in 2013–2014 at various conferences (a collection of posters can be found at Oncomed Pharmaceuticals’ website). Vantictumab has clear pharmacodynamics effects on expression of stem cell and differentiation genes in the tumor, as well as in hair follicles and bones. The initial enthusiasm however has recently diminished when clinical trials were put on hold (see section "Safety of Wnt pathway targeting" below).

Secreted Frizzled-related proteins (sFRPs) have also been in focus as targets for cancer therapy. There are conflicting reports in the literature as to whether sFRPs are antagonists or agonists of Wnt/β-catenin signaling [58–60]. For example, sFRP2 is over-expressed in human angiosarcoma and breast cancer and stimulates angiogenesis via activation of the calcineurin/NFATc3 pathway. Recently, sFRP2 has been assessed as a viable therapeutic target [61]. sFRP2 mAb has been shown to induce anti-tumor and anti-angiogenic effects *in vitro* and inhibit activation of β-catenin and nuclear factor of activated T-cells c3 (NFATc3) in endothelial and tumor cells. sFRP2 mAb treatment of angiosarcoma allografts or MDA-MB-231 breast carcinoma xenografts in nude mice significantly reduced tumors volume. Given that this mAb preferentially accumulates in sFRP2-positive tumors and is long circulating in the blood, it may be a good candidate for clinical studies.

Even more perspective would be an approach combining two or more targeted therapies. In this respect a recent study by Birchmeier and colleagues [62] is of a particular interest. The authors have generated a compound mutant mouse model of aggressive basal breast cancer, combining the activation of β-catenin and HGF (Wnt-Met signaling). They identified the chemokine system CXCL12/CXCR4 as a crucial driver of these tumors. Molecular therapy targeting Wnt, Met and CXCR4 significantly delayed tumor development in mice. Moreover, the gene signature identified in these model tumors was found to be predictive of poor survival in human patients with ER-negative breast cancers. Although the small molecule inhibitors used in this study are only prototypes of the possible future drugs, the outlook is promising. In such a combination therapy, inhibition of the Wnt/β-catenin pathway would stop self-renewal program of CSCs, while suppressing CXCR4 signaling would lead to differentiation of cancer propagating cells.

### Regeneration

**Figure 2:**
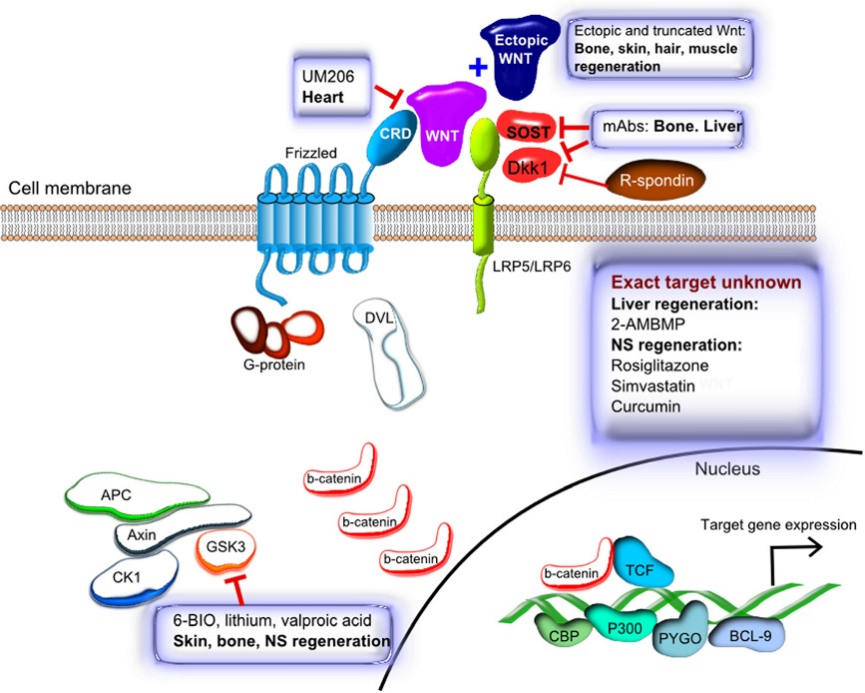
**Schematic representation of the Wnt/β-catenin signaling pathway and the regeneration therapy drug candidates discussed in the paper.** The molecular targets (where known) of the small molecule and antibody-based drug candidates are shown.

The Wnt/β-catenin pathway is known to be essential for stem cell proliferation. While life-threatening in the context of CSCs, this fact underlies the importance of the pathway in regenerative processes in many tissues, such as the liver, brain, muscle, skin and bone. This makes Wnt pathway agonists desirable candidates for regeneration-enhancing therapies, and some have already shown their regenerative effects. We will review the role of different branches of the Wnt pathway (but mostly Wnt/β-catenin) separately for different organs and tissues, paying special attention to the therapeutic potential and successful practical applications of activators of the Wnt signaling (Figure 2 and Table 2).

**Table 2:** **Current status of regeneration-related clinical trials of biologics and small molecules targeting the Wnt/β-catenin pathway**

Name/company	Target	Agent	Conditions	Clinical phase
Wnt3a (China Medical University Hospital)	FZDs	Native Wnt3a	Primary Disease	recruiting
AMG785 (Amgen)	Sclerostin	mAb	Bone Fracture Healing, Osteoporosis	II
Valproic acid (Seoul National University Hospital)	GSK-3β	Valproic acid	Androgenetic Alopecia	II
			Male Pattern Baldness	
AMG 162 (Amgen)	Dkk1	mAb	Osteoporosis	II
HSC (Histogen)	multiple	Wnt7a-containing complex	Androgenetic Alopecia	II

### Liver

The Wnt/β-catenin signaling is known to be involved in liver organogenesis during embryonic development [63] and is active at all stages of the organogenesis, activating cell proliferation through c-Myc and cycD1 and regulating hepatocyte differentiation via an interplay with HNF4, C/EBPa, BMP4, and some other pathways [64, 65].

In the normal adult liver the Wnt/β-catenin pathway is generally inactive, and β-catenin undergoes phosphorylation and subsequent degradation [66]. Nevertheless, re-activation of the pathway has been shown as one of the driving forces essential for liver regeneration, in terms of liver stem cells proliferation and differentiation [67].

In the liver, the role of stem cells is known to be played by adult hepatic progenitor cells also known as oval cells [68]. The oval cells have been shown to participate in regeneration and in a range of human liver diseases, such as HCC. The nature of liver oval cells is bi-potential as they have been shown to differentiate toward both the hepatic and bile ductular epithelial lineages in the liver [69]. It has been demonstrated that active Wnt/β-catenin signaling in the liver occurs preferentially within the oval cell population, and forced over-expression of a constitutively active β-catenin mutant drives expansion of the oval cell population in the regenerating liver [70]. The dual role of the Wnt/β-catenin pathway in regulating the fate of the oval stem cells has been shown by demonstration that Wnt1 can induce their differentiation to hepatocytes in a rat liver regeneration model [71]. This example indicates the tissue-specific importance of certain Wnt ligands as candidates for the use in regenerative medicine.

The importance of Wnt/β-catenin signaling for the liver regeneration has been shown in a series of other observations focusing on different levels of regulation of the Wnt cascade. For example, it has been demonstrated that Wnt-dependent regeneration in the liver can be controlled by an endogenous long non-coding RNA, which activates Wnt/β-catenin signaling by inhibiting expression of Axin. This action of the non-coding RNA leads to enhanced hepatocyte proliferation during liver regeneration [72], showing the potential for the therapeutic use of Wnt pathway-related non-coding regulatory RNAs in the treatment of liver diseases.

Several examples demonstrating the interplay of the Wnt/β-catenin pathway with other signaling cascades during liver regeneration have recently been described. The TGFβ family-related protein SMAD6 is able to suppress the Wnt/β-catenin signaling in the liver acting at the β-catenin-TCF-promoter interaction; down-regulation of SMAD6 leads to increased Wnt-dependent proliferation and self-renewal of the hepatic progenitor cells [73]. The Wnt/β-catenin pathway also interacts with Hedgehog signaling in the liver, being able to stimulate expression of the transcription factor Gli1 which is a downstream effector of the Hedgehog pathway. Gli1 on its turn up-regulates cycD1 causing enhanced hepatocyte proliferation [74].

Given the importance of Wnt/β-catenin signaling for hepatocyte proliferation and differentiation, therapeutic intervention in the Wnt signaling pathway appears as a promising tool for liver regeneration. The first successful attempts have been already made. Liver cells expressing Lgr5, a marker of actively dividing stem cells in Wnt-driven self-renewing tissues, are able to clonally expand *in vitro* from single cells and form transplantable "organoids", retaining many characteristics of the original epithelial architecture, under the influence of a Wnt synergist R-spondin1 which acts as a Dkk1 competitor [75]. Such approaches are paving the way to future *de novo* growth of transplantable organs.

A therapeutic effect of a small molecule Wnt/β-catenin pathway agonist, 2-amino-4-(3,4- (methylenedioxy)benzylamino)-6-(3-methoxyphenyl)pyrimidine (2-AMBMP), has been demonstrated in the hepatic ischemia model in rats. The drug blunted the ischemia-induced elevation of aspartate aminotransferase and alanine aminotransferase levels, increasing cell proliferation rate and decreasing negative effects such as inflammation and apoptosis, and reduced the death rate in general [76]. As small molecules are often a cheap and convenient substitution for natural effector proteins, such the therapeutic approach represents a promising strategy for the liver regeneration therapy.

### Bone

Wnt signaling is known to be a key regulator of bone tissue growth in embryos and in the adult [77]. It is essential for osteocyte formation from stem cells [78] and for bone regeneration after injury or disease [77]. Enhanced Wnt/β-catenin signaling increases bone volume and causes hyperostosis and pathological bone thickening (sclerosing bone dysplasia) [79–82]. Such effects are achieved by stimulating the bone-forming osteoblast activity [83, 84], by inhibiting the bone disassembling osteoclast function [85, 86], and by differentiation of diverse pluripotent stem cells toward osteoblasts [87–89]. It has been reported that a number of components of the Wnt/β-catenin pathway, such as the ligands Wnt4, Wnt5a, Wnt10b, Wnt11, and Wnt13, the receptors FZD_1_, _2_, _4_ and _5_, the co-receptors LRP5 and LRP6, β-catenin, and the Wnt target genes, such as the osteoblast differentiation-associated transcription factor Runx2, are up-regulated in the fracture calyx during bone regeneration [90–94]. It needs be highlighted that not only β-catenin-dependent Wnt signaling takes part in the bone tissue growth, but other branches of the pathway control it as well. It has recently been shown that β-catenin-independent signaling through Wnt5a-FZD_9_ is significantly up-regulated during the early stages of osteoblast differentiation [95] and is reactivated in the regenerating bone, being essential for the fracture healing in a femur osteotomy model in *Fzd*_*9*_^-/-^ mice [96]. The β-catenin-independent Wnt5a signaling has been reported to efficiently promote trans-differentiaon of adipoprogenitors into osteoblasts in culture by suppressing peroxisome proliferator-activated receptor (PPAR)-γ, a key adipogenesis-stimulating transcription factor, and by activating Runx2 [97]. *Wnt5a*^*+/-*^ mice demonstrate decreased trabecular bone mass in the femurs, and mice homozygous for the loss-of-function mutation in *Wnt5a* show truncation of the proximal skeleton and lacking of distal digits [98]. The β-catenin-independent Wnt5a signaling has been also shown to inhibit apoptosis of differentiated osteoblasts and their progenitors, comparable to the anti-apoptotic effects of signaling by Wnt3a and Wnt1 involving β-catenin [99].

Taken together, these data suggest that Wnt signaling modulators could be used as therapeutic agents to stimulate bone formation after injury or disease. Currently two strategies are exploited to find an effective Wnt-based bone regenerative therapy. The first involves reduction of the action of native Wnt signaling inhibitors endogenously present in the organism, which in turn would enhance the intrinsic Wnt activity. In this regard, activation of Wnt signaling by neutralizing antibodies against the Wnt inhibitors Dkk1 [100] and sclerostin [101–104] has been shown to improve bone healing in mice. sFRP1 is also a Wnt inhibitor and a promising target for bone regeneration therapy [105].

Another way to promote Wnt signaling in order to boost the bone tissue growth is a direct use of exogenous Wnts or other pathway agonists. Although native Wnt proteins are hard to use due to their low solubility, a successful attempt has been recently made with liposome-loaded Wnt3a, which upon application to the injury site has caused a ca. 3.5-fold increase in the bone regeneration rate in mice [106]. A clinical trial, sponsored by the China Medical University Hospital (Taiwan), is being initiated to study the osteogenic effects of human mesenchymal stem cells enhanced by Wnt3a charged on hydroxyapatite nanoparticles ( http://clinicaltrials.gov/show/NCT01323894).

Stimulation of murine bone regeneration has been also obtained by application of LiCl [92], and similar effects on rat bone regeneration have been observed using Li_2_CO_3_ [107]. Li^+^ is a well-known inhibitor of GSK3β – a key enzyme targeting β-catenin for proteosomal degradation – and thus activator of the Wnt pathway; however, it is less specific than Wnt proteins or other pathway agonists and therefore may cause more side effects. In general it can be stated that Wnt signaling in the bone tissue is well studied, leading to a big variety of possible artificial modulations of the pathway to improve bone healing and to treat bone diseases.

### Skeletal muscle

The role of Wnt/β-catenin signaling in the skeletal muscle remains controversial. In the adult, the β-catenin-dependent Wnt pathway has been suggested to control myogenic lineage progression by limiting Notch signaling and thus promoting differentiation [108, 109], in particular through Myf5 and MyoD growth factors [110]. Other data demonstrate that Wnt/β-catenin signaling in the adult tissue, e.g. through Wnt1 and Wnt3a, promotes only slow myofiber types generation and inhibits myogenesis in general [111]. But the role of Wnt proteins in regenerative processes in the skeletal muscle differs from that in most other tissues, due to increased significance of the β-catenin-independent Wnt activity. It has been shown that Wnt7a, signaling through its receptor FZD_7_, is capable of activation of distinct pathways at different stages of myogenesis. First, the Wnt7a-FZD_7_ interaction leads to activation of the Wnt-PCP (planar cell polarity) pathway which is responsible for symmetric expansion of satellite cells in the muscle tissue [112] – a small sub-population of muscle cells that are capable of self-renewal and act as muscle stem cells during regeneration [113]. Over-expression of Wnt7a enhances muscle regeneration by means of increasing the satellite cell number, whereas muscles with down-regulated Wnt7a exhibit a significant decrease in satellite cell numbers, impairing the regeneration capacity. At this level of action, Wnt7a does not affect the growth or differentiation of myoblasts. Next, stimulation of FZD_7_ by Wnt7a in differentiated myofibers directly activates the Akt/mTOR growth pathway through Gαs and PI3K, thereby inducing myofiber hypertrophy [114]. Thus Wnt7a serves as a muscle growth-promoting factor at two levels, and at both it acts independently from β-catenin. Recently a third level of Wnt7a activity in regenerating muscles has been described, where the Wnt7a/FZD_7_ interactions have been shown to increase the polarity and directional migration of murine and human myogenic progenitors through activation of Dvl2 and the small GTPase Rac1, resulting in improved muscle strength [115].

Taken together, these findings identify Wnt7a as an important drug candidate against muscle wasting diseases like sarcopenia, cachexia or muscular dystrophies, and for improvement of stem cell-based muscle regenerative therapy. Therapeutic potential of Wnt7a has indeed been tested in several systems, like the mouse model of Duchenne muscular dystrophy, where the Wnt7a treatment stimulated satellite cell expansion and myofiber hypertrophy and even led to a significant increase in muscle strength. Furthermore, Wnt7a decreased the level of contractile damage, most likely by inducing a shift in the fiber type toward the slow-twitch [114]. In other experiments a short treatment with Wnt7a notably increased muscle tissue dispersal and engraftment, significantly improving the muscle function [115].

Interestingly, a truncated Wnt7a variant consisting of only 137 C-terminal amino acids and devoid of the conserved palmitoylation sites has recently been shown to preserve the full biological activity of the native protein in skeletal muscles [116]. It retained the capability of interaction with its receptor FZD_7_ and stimulation of symmetric expansion of satellite stem cells through the PCP pathway, as well as induction of myofiber hypertrophy by signaling through the AKT/mTOR pathway [116]. This finding is of a special importance for the Wnt7a-based muscle therapy, because natural, palmitoylated Wnt proteins are hard to use in medical and biotechnological applications due to their large size, low productivity and poor solubility. Evidence that truncated Wnts may in some cases retain the therapeutic potential may be a first step to production of active Wnt-related peptides, easily applicable to muscle disease treatment.

### Skin and hair follicles

In the skin, β-catenin-dependent Wnt signaling is one of the dominant pathways regulating the patterning and determining the fate of embryonic and adult stem cells during their differentiation, as well as subsequently controlling the function of differentiated skin cells [117]. An important element of the skin are hair follicles, whose morphogenesis also depends on Wnt, along with Shh, Notch, BMP and other signaling pathways interacting with each other. The Wnt/β-catenin pathway is a key player during hair follicle induction, acting through the EDA/EDAR/NF-κB signaling [118].

There is evidence pointing out an involvement of Wnt signaling in the skin repair and hair follicle regeneration. For example, a Wnt synergist R-spondin2 promotes cell proliferation in the adult epidermis [119] which is directly linked to the skin wound healing rate. Wnts also act as niche signals for skin stem cells located in the bulge region of the hair follicles [120, 121]. Although the effects of Wnt/β-catenin signaling were previously regarded to be opposite on the epidermal and the hair follicle stem cells [122], it has recently been shown that β-catenin-dependent signaling actually promotes proliferation of these both stem cell populations, thus being a driving force for regeneration of both the skin and hairs [117, 123]. Interfollicular epidermal stem cells have been shown to express the Wnt target gene Axin2 and to require Wnt/β-catenin signaling for proliferation, producing autocrine Wnts as well as long-range secreted Wnt inhibitors, thus suggesting an autocrine mechanism of stem cell self-renewal in the epidermis. These cells are able to promote skin wound healing, with no demand for a quiescent stem cell subpopulation [117]. In another work, experiments on β-catenin deletion and LRP5/6 inhibitor Dkk1 over-expression have indicated a necessity of Wnt/β-catenin signaling for follicular stem cell proliferation; the same work confirms the data on the Wnt-dependent renewal of the inter-follicular epidermis [123]. Activation of β-catenin specifically within murine hair follicle stem cells has been found sufficient to induce hair growth independently of mesenchymal dermal papilla niche signals normally required for the hair regeneration [124].

Artificial up-regulation of Wnt/β-catenin signaling in regenerating hair follicles through adenoviral infection of mice with Wnt10b has led to an increase in the size of regenerating follicles and to excessive proliferation of follicle cells. The observed effects were reduced when the mice were co-infected with Dkk1 and Wnt10b [125]. These findings suggest that ectopic Wnt10b and Dkk1 can be used to modulate the follicle size and proliferation during hair regeneration.

Another example of effective use of external Wnt treatment to improve the skin wound healing rate is the injection of Wnt3a-carrying liposomes into the skin of injured mouse ears. The liposomal Wnt3a has demonstrated the ability to enhance Wnt signaling in the damaged tissue and to dramatically improve the wound healing rate [126]. Hair growth (seen as hair follicle progression from telogen to anagen and by overall up-regulation of hair induction-related genes) could also be enhanced in mice upon treatment with Wnt1-conditioned media [127]. Interestingly, there are clinical data showing that ectopic application of Wnt7a as a component of a growth factor cocktail enhances hair growth, possibly suggesting that Wnt7a could act in a β-catenin-dependent way in this case [128].

Finally, more robust ways to up-regulate Wnt/β-catenin signaling have been successfully applied to skin and hair regeneration. Valproic acid, an inhibitior of GSK3β, has shown its efficacy in boosting the cutaneous wound repair in mice [129] as well as promoting hair growth in cell culture [130] and inducing hair regeneration in murine systems [131] and in clinical trials [132]. Along with the bone, the skin and hair regeneration is the field where the Wnt-based therapy is well advanced, as compared to other organs and tissues.

### Neuroprotection and Alzheimer’s disease

Wnt signaling is actively involved in neurogenesis and modulation of the synaptic function in the adult [133]. Further, association of the Aβ-peptides and Alzheimer’s disease etiology in general with the Wnt signaling-induced processes has been reported many times [134, 135]. The Aβ-peptides have been shown to directly inhibit the Wnt/β-catenin cascade by interacting with FZDs and impairing the normal activation of the pathway [136]. Such impairment can in turn lead to synaptic degeneration. On the other hand, addition of exogenous Wnt3a or LiCl activating the Wnt/β-catenin pathway has been shown to prevent the Aβ-peptide-mediated synaptic degeneration and cell death in a neuronal cell culture [137]. It has also been shown that neuroprotective effects of Wnt3a are mediated by the receptor FZD_1_ [138]. Recent studies confirm the importance of the Wnt/β-catenin signaling as an anti-Alzheimer acting force, as activation of the cascade using Bromoindirubin-30-Oxime (6-BIO), an inhibitor of GSK3β, protects hippocampal neurons from Aβ-oligomers and reduces the rate of neuronal apoptosis [139]. Moreover, the same study suggests that the β-catenin-independent Wnt5a-mediated Ca^2+^-dependent signaling could modulate mitochondrial dynamics and prevent the changes induced by the Aβ-peptide oligomers in mitochondrial fission and fusion usually present in neurodegenerative diseases [139].

Other small molecule Wnt/β-catenin signaling agonists have also demonstrated an ability to increase adult neurogenesis or inhibit the neurodegenerative effects of the Aβ-peptides. Simvastatin has been shown to synergize with Wnt/β-catenin signaling *in vivo* and *in vitro*, enhancing it through depletion of isoprenoid synthesis (which is involved in the regulation of membrane-located proteins like small GTPases) and improving the rate of adult hippocampal neurogenesis, making it a potential neuroprotective drug [140]. In a mouse model of Alzheimer’s, treatment with LiCl reduced amyloid-induced memory impairment and decreased Aβ-peptide aggregation. These effects were paralleled by stabilization of β-catenin [141].

Another interesting example of small molecule agonists of Wnt/β-catenin signaling able to act in the brain is curcumin. Cucrumin-coated nanoparticles could activate the Wnt/β-catenin pathway in rat brains and reverse learning and memory dysfunctions in the Aβ-induced rat model of the Alzheimer’s disease by stimulation of neurogenesis. *In silico* molecular docking studies suggest the possible mechanisms of curcumin action through interaction with Wnt antagonists Wif-1, Dkk, and the β-catenin inhibitor GSK3β [142]. These observations make the therapies based upon the Wnt-cascade activation a promising way to treat the Alzheimer’s disease and other neurodegenerative diseases.

However, in case of the brain tissue, over-activation of Wnt pathways can also be counterproductive in therapeutic applications, as Wnts are known to stimulate pro-inflammatory processes in microglia, thus complicating the Alzheimer’s disease treatment. An increase in Wnt/β-catenin signaling has been observed in microglia of mice with Alzheimer’s-like pathology; in cultured microglia, treatment with Wnt3a activated the β-catenin-dependent pathway and led to increased expression of pro-inflammatory genes [143]. Experiments on the axon injury model in mice have demonstrated that down-regulation of β-catenin-dependent signaling by a tissue-specific gene knockout in oligodendrocyte precursor cells facilitates axonal regeneration in damaged tissue and reduces the glial scarring [144]. Interestingly, β-catenin-independent Wnt5a signaling is also implicated in microglial pro-inflammatory transformation via the ERK1/2 pathway [145]. Such controversial involvement of distinct Wnt pathway branches in regeneration and inflammation of the neural tissue will require a more detailed study of the interplay of different signaling pathways and a very accurate application of Wnt signaling activators in case of future therapeutic Alzheimer’s disease treatment.

### Discussion on regeneration

Generally, the regeneration processes in most tissues are regulated by re-activating the Wnt/β-catenin signaling, which leads to stem cell proliferation and/or differentiation, although in some cases β-catenin-independent Wnts take on the leading role, as it is the case with the skeletal muscle. In spite of a complex interplay of Wnt signaling with other signaling cascades, straightforward approaches like ectopic treatment of the injured organs or tissues with Wnt agonists have shown their effectiveness in different tissues, improving the regeneration rate. Combining these findings with possibilities to further enhance therapeutic potential of Wnts through viral or liposomal delivery or by engineering of small Wnt-related peptides allows us to look optimistically towards the direct use of Wnt cascade agonists in regenerative medicine. In addition to application of the truncated Wnt7a in the muscle, short peptide analogs of Wnt5a or their more stable formylated derivatives have shown to mimic the biological activity of the native Wnt5a protein by blocking cell migration in breast epithelium [52, 53]. Structure-assisted design [44, 146] and directed protein evolution approaches [147] can help in generation of novel Wnt-derived pro-regenerative agents. Other approaches are the indirect methods to enhance intrinsic Wnt activity in the injured or diseased tissue and have also proven their applicability. For example, down-regulation of the natural Wnt inhibitors has been shown to work well in aiding the bone repair. Several regenerative therapeutic approaches targeting the Wnt/β-catenin pathway in the skin and the bone are already in clinical trials (Table 2).

Liver, brain, skin and bones are the well-studied organs for Wnt/β-catenin regenerative applications, but they do not limit the potential of possible targets for the Wnt-based regeneration therapy. It is known that Wnt/β-catenin signaling is also involved in angiogenesis [148], indicating that modulation of this pathway might be used as an instrument to treat cardiovascular diseases including myocardial infarction [149] or target angiogenesis in tumors. Practical attempts in this direction have already been made: recent studies indicate that UM206, a small peptide homolog of Wnt3a/Wnt5a capable of blocking FZD signaling, can improve heart tissue regeneration after infarction [150].

Approaches for the future regenerative medicine do not stop just at the increasing of the natural regeneration rate. A highly desired goal is to develop technologies of tissue engineering and of growth and transplantation of artificial organs. Although no complete functional organs ready for transplantation have been grown so far, first efforts have already been made in this direction. Such complex tasks would involve multiple steps and demand compound cocktails of different growth factors, where Wnt signaling activators are essential components in most cases. A successful attempt to grow an optic cup (retinal primordium) structure from a three-dimensional culture of mouse embryonic stem cell aggregates has been performed, which involved a treatment with Wnt/β-catenin pathway agonists at certain steps [151]. As a conclusion, it can be claimed that development and production of highly active and tissue-specific agonists of the Wnt/β-catenin signaling, either peptide-based or small-molecule analogs, is a goal of high importance for regenerative medicine in the next decades.

### Safety of Wnt pathway targeting

As exemplified above, the Wnt pathways are involved not only in many developmental processes but also in the maintenance of adult tissue homeostasis. Thus a careful safety assessment of drugs directly or non-directly targeting the Wnt signaling is required. It is clear that a general inhibition of the Wnt/β-catenin pathway is potentially unsafe, mainly because of: (i) broad Wnt/FZD expression pattern; (ii) the role of the pathway in the maintenance of the differentiated epithelium and its interaction with mesenchymal cells; (iii) its involvement in stem cell pluripotent state maintenance; (iv) its role in bone homeostasis. A recent review [152] provides a very detailed discussion of safety concerning targeting of the Wnt pathways, both in regenerative medicine and oncology. Here we would like to provide only a few illustrations highlighting this safety issue – as well as the issue of difficulty in predicting whether a certain Wnt-targeting drug candidate would or would not have particular side effects.

The first example is about the FZD co-receptor antagonists. Blockade of Wnt/β-catenin signaling by adenovirus-mediated expression of Dkk1 (a natural LRP5/6 antagonist) in mice has been shown to suppress epithelium proliferation in small intestine and colon, accompanied by progressive architectural degeneration with the loss of crypts, villi, and glandular structure by 7 days [153]. Further, ectopic Dkk1 expression led to a complete failure of hair follicule formation in adult mice [154]. In contrast to that *in vivo* administration of an LRP6 antagonist Mesd markedly suppressed growth of MMTV-Wnt1 tumors without causing undesirable side effects [155].

Another example concerns the potential of using Wnt proteins as drugs. Indeed, some Wnts have tumor suppressing activity. Wnt5a, for example, is often viewed as a "non-canonical" Wnt capable of antagonizing the Wnt/β-catenin signaling and has a role in limiting B-cell proliferation and functions as a tumor suppressor in hematopoietic tissue [156]. Mice hemizygous for Wnt5a develop clonal myeloid leukemias and B cell lymphomas and display loss of Wnt5a function in tumor tissues. On the other hand, expression of Wnt3a (a prototypical activator of the Wnt/β-catenin signaling and thus a presumed pro-oncogenic factor) in mouse melanoma model decreased the proliferation of the tumor and suppressed the metastasis [157]. Therefore whether the application of a certain Wnt will be anti- or pro-oncogenic may be highly context-depending.

In many cases, the side effects whose presence or seriousness has been ruled out during pre-clinical studies appear during clinical trials, despite all pre-cautionary measures. For example, the enthusiasm initially shown for both of Oncomed Pharmaceuticals’ Wnt pathway biologics, vantictumab and OMP-54F28, diminished when clinical trials for both were put on hold. The quoted reasons for halting patient enrollment dosing are bone-related side-effects ranging "from mild to moderate" ( http://www.reuters.com/article/2014/06/13/us-oncomed-study-idUSKBN0EO1B920140613). These were observed in 8 out of 63 (13%) patients treated with vantictumab and in 2 out of 41 (5%) with OMP-54F28. Since the increase in bone turnover has been stated as one of the vantictumab pharmacodynamics effects, it is logical to assume that the mentioned percentage of patients developed osteoporosis. Lowering the dosage and frequency of the drugs might neutralize such side effects – but this could also have a negative impact on the drug efficacy.

The root of the current problem with vantictumab in particular may lie in the initial design of the drug molecule. It binds to 5 out of 10 FZD family members *in vitro* and potentially even more *in vivo*. However, not all of the FZDs that bind to vantictumab are over-expressed or over-activated in the solid tumors included in the clinical study. Conversely, only some FZD members may be critical for bone remodeling. A FZD mAb with less broad specificity could still have been effective, inhibiting signaling originating from pro-oncogenic FZDs (or just one FZD) while not affecting the FZDs indispensable for osteogenesis. These considerations highlight perhaps the most important issue in targeting the Wnt pathways – safety is likely to result from higher specificity in targeting a particular sub-pathway implicated in a certain pathogenic condition, rather than bluntly suppressing all or most of the sub-pathways [158, 159].

## Conclusion

As discussed above, Wnt signaling plays important functions in cancer progression and tissue regeneration. Ironically, what is therapeutically good for one (activation of the pathway in regeneration) is bad in for the other (anti-cancer treatment). Thus, extreme care must be taken when developing Wnt-stimulating pro-regenerative drugs, to exclude the risks of cancer complications. This demand appears not un-achievable through design of highly specific and/or local activators of the Wnt pathways. On the other hand, the expected side effects in targeting the Wnt/β-catenin pathway in anti-cancer treatments are myelo- and gastrointestinal suppression, exactly due to the adverse effects of anti-Wnt treatments on proliferation of hematopoetic and intestinal stem cells, as well as progenitor cells in other organs. Here again, a hope is for designing specific agents targeting the Wnt/FZD sub-pathways activated in a given cancer, rather than blocking the Wnt/β-catenin signaling altogether [159]. In this regard, attacking higher ‘floors’ of the signaling hierarchy – i.e. the ligands and receptors – appears especially promising [48, 158]. It is important to remember that the Wnt pathways must be fine-tuned for the normal physiology control. As recent works indicate [47], even moderate attenuation of Wnt signaling can eliminate its carcinogenic potential. Therefore it is possible to keep the "good" physiological level of Wnt signaling by finding the right target and a right tool to act on this target in a desired mode. Perhaps the fact that Wnt signaling in tumor tissues is exacerbated relative to normal tissue as well as a potential of normal tissue to recover could provide the safe window of Wnt inhibition in therapy.

## Abbreviations

AML: Acute myeloid leukemia
2-AMBMP: 2-amino-4-(3,4- (methylenedioxy)benzylamino)-6-(3-methoxyphenyl)pyrimidine
6-BIO: Bromoindirubin-30-Oxime
CSC: Cancer stem cell
Dvl: Dishevelled
FZD: Frizzled
GPCR: G-protein coupled receptor
HCC: Hepatocellular carcinoma
mAb: Monoclonal antibody
PCP: Planar cell polarity
sFRP: Secreted Frizzled-related protein
siRNA: Small interfering RNA
TNK: Tankyrase.
